# The tricalbin family of membrane contact site tethers is involved in the transcriptional responses of *Saccharomyces cerevisiae* to glucose

**DOI:** 10.1016/j.jbc.2024.107665

**Published:** 2024-08-10

**Authors:** Philipp Schlarmann, Keiko Sakuragi, Atsuko Ikeda, Yujia Yang, Saku Sasaki, Kazuki Hanaoka, Misako Araki, Tomoko Shibata, Muneyoshi Kanai, Kouichi Funato

**Affiliations:** 1 Graduate School of Integrated Sciences for Life, Hiroshima University, Higashi-Hiroshima, Japan; 2 National Research Institute of Brewing, Higashi-Hiroshima, Japan

**Keywords:** membrane contact sites, signaling, glucose metabolism, gene regulation, amino acid, yeast

## Abstract

Cellular organelles maintain areas of close apposition with other organelles at which the cytosolic gap in between them is reduced to a minimum. These membrane contact sites (MCS) are vital for organelle communication and are formed by molecular tethers that physically connect opposing membranes. Although many regulatory pathways are known to converge at MCS, a link between MCS and transcriptional regulation—the primary mechanism through which cells adapt their metabolism to environmental cues—remains largely elusive. In this study, we performed RNA-sequencing on *Saccharomyces cerevisiae* cells lacking tricalbin proteins (Tcb1, Tcb2, and Tcb3), a family of tethering proteins that connect the endoplasmic reticulum with the plasma membrane and Golgi, to investigate if gene expression is altered when MCS are disrupted. Our results indicate that in the *tcb1*Δ*2*Δ*3*Δ strain, pathways responsive to a high-glucose environment, including glycolysis, fermentation, amino acid synthesis, and low-affinity glucose uptake, are upregulated. Conversely, pathways crucial during glucose depletion, such as the tricarboxylic acid cycle, respiration, high-affinity glucose uptake, and amino acid uptake are downregulated. In addition, we demonstrate that the altered gene expression of *tcb1*Δ*2*Δ*3*Δ in glucose metabolism correlates with increased growth, glucose consumption, CO2 production, and ethanol generation. In conclusion, our findings reveal that tricalbin protein deletion induces a shift in gene expression patterns mimicking cellular responses to a high-glucose environment. This suggests that MCS play a role in sensing and signaling pathways that modulate gene transcription in response to glucose availability.

The interior of living cells is characterized by an intricate network of membrane-enclosed organelles. Membrane contact sites (MCS), areas where organelles physically interact over a short ∼30 nm wide cytosolic gap, serve as hubs for intracellular communication and metabolite distribution. MCS mediate numerous processes, including organelle-related functions such as membrane dynamics, organelle quality control, trafficking, and biogenesis, as well as those that affect the broader cell's equilibrium, such as calcium signaling, reactive oxygen species signaling, lipid signaling, lipid metabolism, stress responses, and apoptosis (1, 2, 3, 4, 5, 6, 7). The dysregulation of MCS has consequently been implicated in the development of various diseases including neurological diseases and cancer (8, 9).

The tricalbin proteins (Tcb1, Tcb2, and Tcb3) are an evolutionary conserved family of endoplasmic reticulum (ER) tethering proteins that connect with four other tethers (Ice2, Ist2, and Scs2/22) the ER with the plasma membrane (PM) (10). Our recent studies have revealed that tricalbins additionally function in MCS formation and nonvesicular ceramide transport between the ER and the Golgi apparatus (11), thereby connecting three organelles by two MCS. Moreover, we have reported that tricalbin deficient cells exhibit abnormal organelle morphology characterized by an increased number of lipid droplets (11) and fragmented vacuoles (12). These widespread effects on other organelles suggest an important role of tricalbins for cellular homeostasis. Under physiological conditions, cellular homeostasis faces ongoing challenges imposed by fluctuating environmental factors, such as changes in temperature, osmolarity, or nutrient availability (13, 14, 15, 16). Among these, the response to variations in glucose and nitrogen availability has been extensively characterized, with both responses modulating the transcription of numerous genes. The response to glucose depletion involves the activation of cytosolic and PM-resident sensors for intracellular and extracellular glucose levels, respectively, and the activation of three major signaling pathways (Snf3-Rgt2; cAMP-PKA; and Snf1-Mig1-Hxk2) that alter gene expression to allow the appropriate metabolic pathways to function properly (15, 17, 18, 19). Similarly, three partially interconnected pathways are known to respond to variations in nitrogen abundance and quality, the general amino acid control pathway, the nitrogen catabolite repression pathway and the target of rapamycin complex 1 (TORC1) pathway (15). Upon amino acid limitation, these pathways mediate gene expression to promote amino acid synthesis, utilize alternative nitrogen sources, and adjust cellular growth, respectively. Likewise, MCS are known to dynamically respond to environmental cues such as glucose starvation and ER stress (20, 21, 22, 23), but whether MCS play an important role in transcriptional regulation in response to environmental changes is not well understood. Notably, a recent study investigated changes in gene expression upon loss of all seven ER-PM tethers and reported a function of ER-PM contacts in the negative transcriptional regulation of the environmental stress response (24). However, it remains so far unanswered how each of the seven tethering proteins at ER-PM MCS contribute to changes in gene expression.

To identify the specific role of the three tricalbin proteins for transcriptional regulation, we analyzed gene expression in WT and *tcb1*Δ*2*Δ*3*Δ strains using RNA sequencing. We found that upon loss of tricalbins, genes involved in amino acid synthesis and glucose metabolism were among the strongest upregulated. Fluorescent microscopy experiments using GFP-tagged glucose transporter Hxt1 and Hxt2 and a fluorescent glucose uptake probe revealed increased expression of low-affinity transporter Hxt1, decreased expression of high-affinity transporter Hxt2 as well as reduced glucose uptake in the *tcb1*Δ*2*Δ*3*Δ mutant. Moreover, we showed increased growth, glucose consumption, CO_2_ production, and ethanol generation in *tcb1*Δ*2*Δ*3*Δ, indicating an adaption of metabolic pathways as if cells are subjected to a high glucose environment and thus a potential role of tricalbins in glucose sensing and signaling.

## Results

### RNA sequencing of *tcb1*Δ*2*Δ*3*Δ revealed strongest changes in amino acid synthesis and mating-related genes

**Figure 1:**
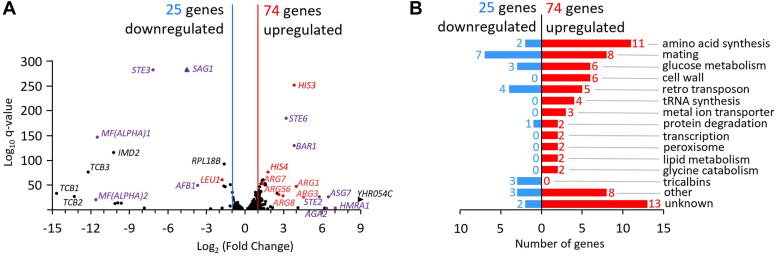
**Altered gene expression in *tcb1*Δ*2*Δ*3*Δ.***A*, volcano plot of differently expressed genes, WT *versus tcb1*Δ*2*Δ*3*Δ from RNA sequencing experiments. Only data from genes with FDR adjusted *p* value < 0.1 is shown. *Red* and *blue lines* indicate thresholds of upregulated and downregulated genes, respectively, with significant thresholds set at Log_2_ fold change over two. Genes of interest are highlighted and categorized in *purple* (mating), *red* (amino acid synthesis), and *black* (other). *Arrow heads* indicate genes outside of the graph (*SAG1* and *YHR054C*, see Table S2). *B*, categorization of significantly upregulated and downregulated genes identified in (*A*) based on their function. FDR, false discovery rate.

To examine mRNA expression levels in the WT and *tcb1*Δ*2*Δ*3*Δ strains, total RNA was extracted from yeast cells cultured in YPD (1% yeast extract, 2% peptone, and 2% glucose) to exponential phase, and mRNA sequencing analysis was performed by contract. mRNA sequencing analysis revealed that among the 5,796 genes analyzed, 74 genes in the *tcb1*Δ*2*Δ*3*Δ strain showed a predominant 2-fold or greater increase in mRNA level, and 25 genes showed a predominant decrease of half or less (Fig. 1*A*). RNA sequencing data were visualized as a volcano plot, highlighting that genes with the highest q-values and fold-change values, whether positive or negative, were predominantly associated with mating. Furthermore, among the most highly upregulated genes, 11 were linked to amino acid biosynthesis, including five related to arginine synthesis and two associated with histidine synthesis. Proteins encoded by all genes identified in Figure 1*A* were classified according to their function in more detail (Fig. 1*B* and Table S2). The most abundant increased genes were those related to amino acid biosynthesis, followed by those related to mating, glucose metabolism, and cell wall. Genes related to mating presented the highest total number of downregulated genes (7) compared to other functional categories, yet the count of upregulated genes was even higher (8). Since amino acid synthesis genes were the strongest one-sided affected group, we continued to investigate other genes involved in amino acid synthesis.

### Gene expression of amino acid synthesis, uptake, and secretion genes is altered in *tcb1*Δ*2*Δ*3*Δ

**Figure 2:**
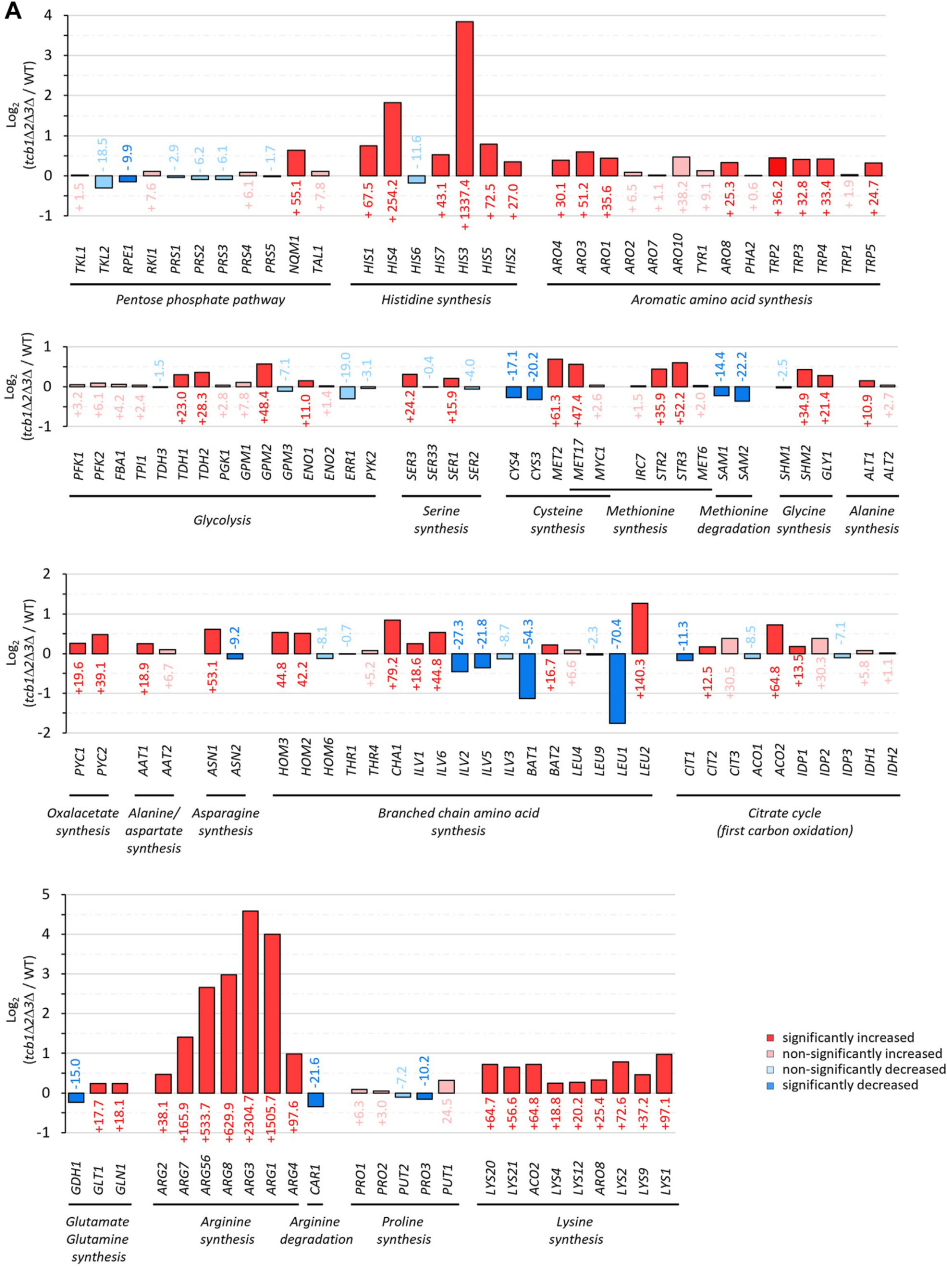

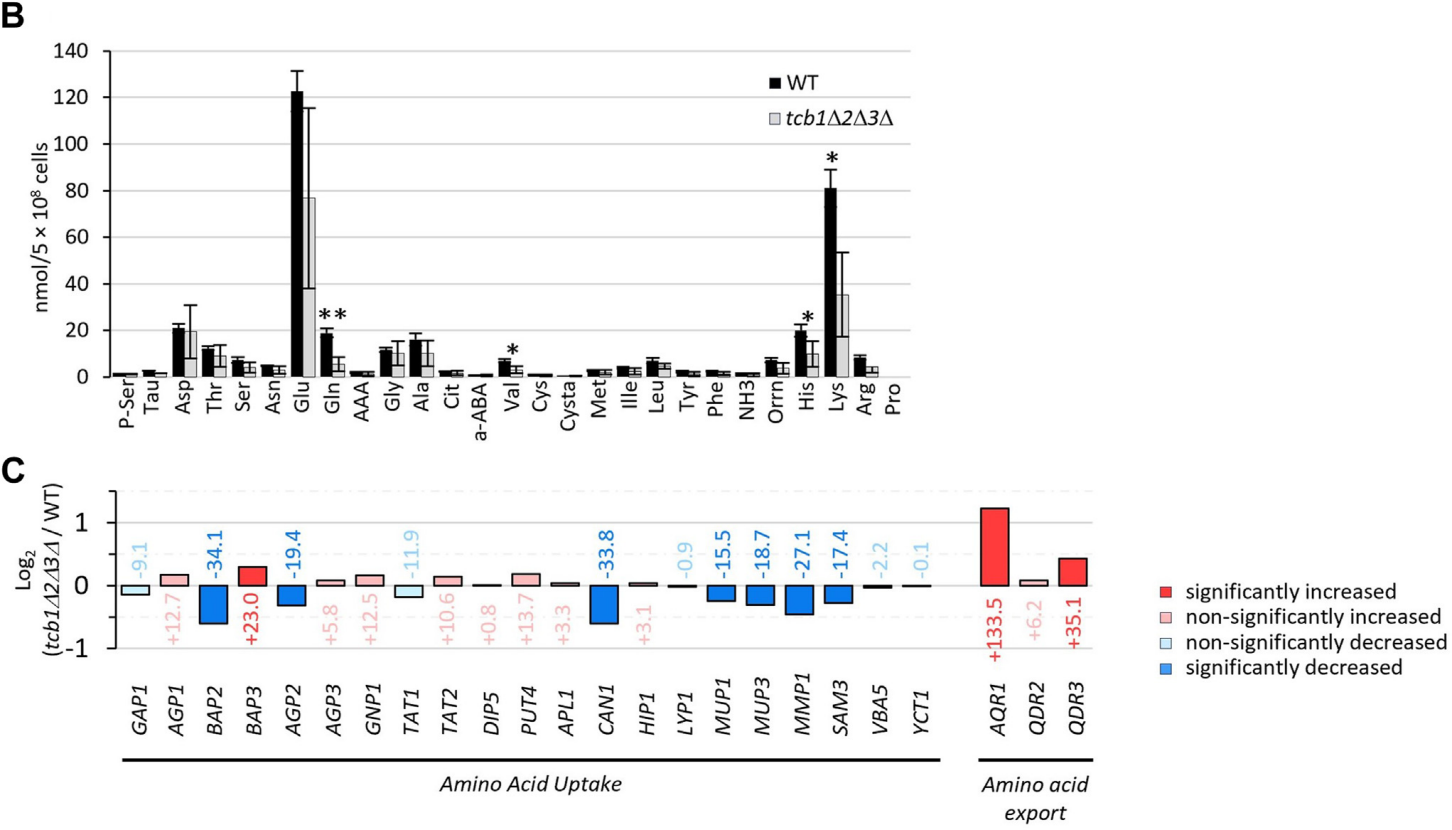
**Altered synthesis and uptake of amino acids in *tcb1*Δ*2*Δ*3*Δ**. *A* and *C*, relative expression levels of genes involved in amino acid synthesis (*A*) and transport (*C*) between WT and *tcb1*Δ*2*Δ*3*Δ depicted as Log_2_ fold change and percent value. Color of genes indicate significant upregulation (*p* < 0.05, *dark red*), nonsignificant upregulation (*p* > 0.05, *light red*), significant downregulation (*p* < 0.05, *dark blue*) and nonsignificant downregulation (*p* > 0.05, *light blue*). Amino acid synthesis genes are arranged based on their sequential order in metabolism and transport genes are arranged from low to high substrate specificity. *B*, amino acids of WT and *tcb1*Δ*2*Δ*3*Δ from whole cell fraction. Results are the mean ± SD of three independent experiments.

Nearly all amino acid synthesis pathways, including those pivotal to produce amino acid precursors such as glycolysis, oxaloacetate synthesis, and the citrate cycle up to the initial carbon oxidation step, showed upregulation (Figs. 2*A* and S1). The most substantial increases were observed in the synthesis of arginine, histidine, lysine, methionine, and aromatic amino acids. Notably, genes responsible for degradation steps, such as *CAR1* for arginine or *SAM1* and *SAM2* for methionine, were significantly downregulated. The only pathways exhibiting a considerable number of both upregulated and downregulated genes were those involved in cysteine, proline, glutamate, and branched chain amino acid synthesis.

We next asked, whether the increases in amino acid gene expression are reflected by the intracellular amino acid concentration and measured total amount of amino acids in WT and *tcb1*Δ*2*Δ*3*Δ. The result showed that the intracellular concentration of specific amino acids is either unchanged or decreased in the *tcb1*Δ*2*Δ*3*Δ, with significant decreases in glutamine, valine, histidine, and lysine (Fig. 2*B*). To investigate the potential reasons for the low amino acid concentration in the *tcb1*Δ*2*Δ*3*Δ strain, we examined the expression of amino acid importers and exporters. The expression of most amino acid and nitrogen importers was significantly reduced, particularly those with high substrate specificity, such as the arginine importer *CAN1* (Fig. 2*C*). In contrast to reduced amino acid uptake, the expression of *AQR1*, *QDR2*, and *QDR3*, membrane transporters involved in amino acid secretion (25), was significantly increased in *tcb1*Δ*2*Δ*3*Δ (Fig. 2*C*). Consequently, the decrease in intracellular amino acid levels in the *tcb1*Δ*2*Δ*3*Δ strain might be attributed to a combination of reduced amino acid uptake and increased amino acid secretion.

### Expression and PM-localization of arginine importer Can1 is reduced in *tcb1*Δ*2*Δ*3*Δ

**Figure 3:**
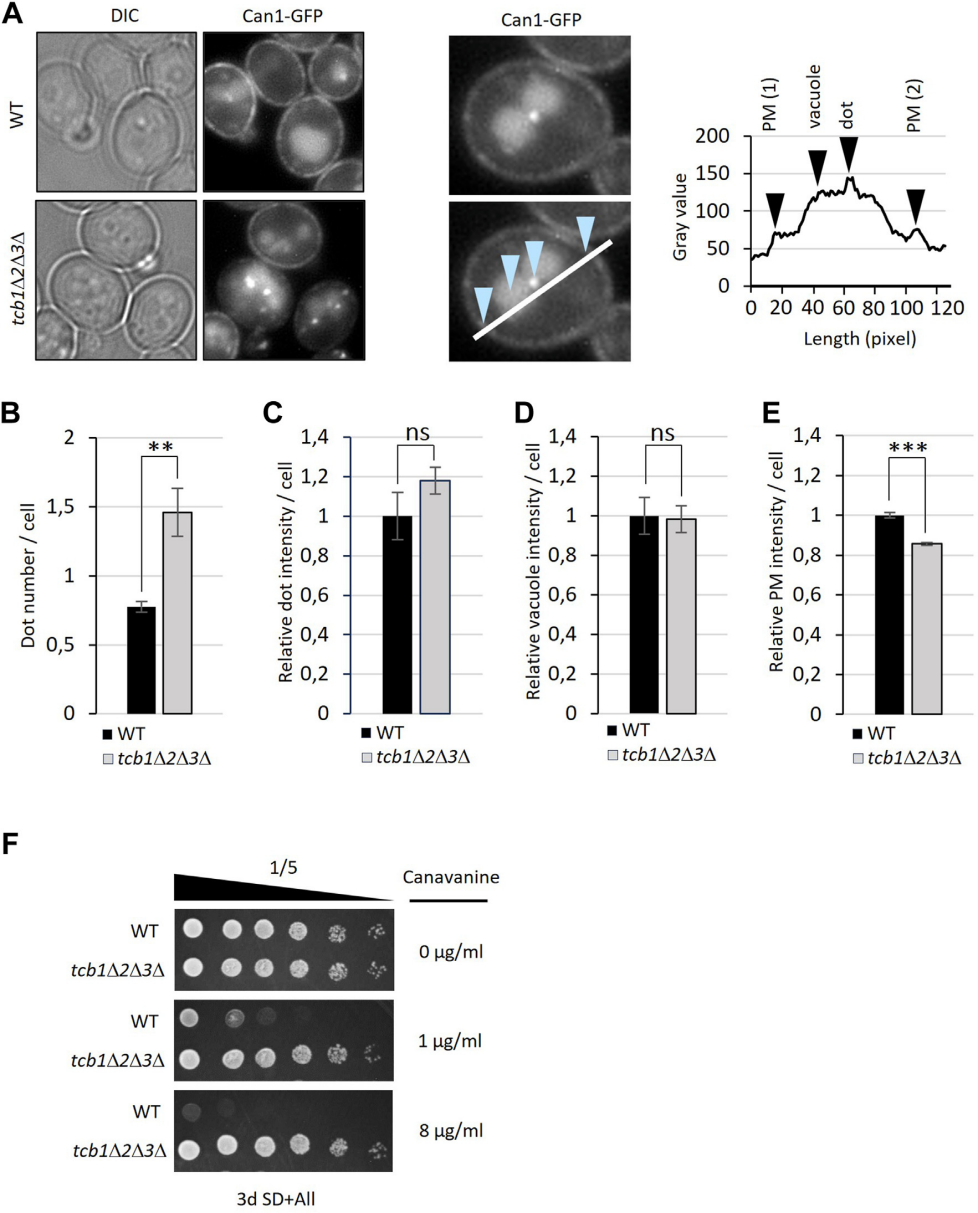
**Localization of Can1 to the PM and canavanine uptakearereduced in *tcb1*Δ*2*Δ*3*Δ.***A*, images of WT and *tcb1*Δ*2*Δ*3*Δ cells expressing endogenously tagged *CAN1-GFP*. Cells were grown in YPD overnight to exponential phase, washed with SD media, and subjected to fluorescence microscopy. Right images and graph show the background adjusted Can1-GFP fluorescence intensity on a line profile. Values for peak PM intensity on two locations, peak vacuole intensity and peak dot intensity were obtained from 100 line profiles in three independent experiments. *B*, average number of cytosolic Can1-GFP dots/cell in WT and *tcb1*Δ*2*Δ*3*Δ. Significance was tested using unpaired *t* test, ∗∗*p* < 0.01. *C*, *D*, and *E*, average fold-change in *tcb1*Δ*2*Δ*3*Δ strain compared to WT of Can1-GFP dot intensity (*D*), peak vacuole intensity (*E*) and peak PM intensity. All graphs show mean values ± SD from three independent experiments. Significance was tested using unpaired *t* test, ns>0.05, ∗∗∗*p* < 0.001. *F*, canavanine resistance of *tcb1*Δ*2*Δ*3*Δ. Fivefold serial dilution of WT and *tcb1*Δ*2*Δ*3*Δ strains on SD plates containing canavanine at the indicated concentrations. Picture shows one out of three independent experiments. PM, plasma membrane; YPD, 1% yeast extract, 2% peptone, and 2% glucose.

To test if the reduction of *CAN1* messenger RNA translates to a real reduction in protein level, we observed endogenously GFP-tagged Can1 by fluorescent microscopy. Can1-GFP localized in both WT and in *tcb1*Δ*2*Δ*3*Δ at the PM, inside the vacuole and in cytosolic and vacuole-adjacent dots that are most likely endosomes. This is consistent with previous reports showing that Can1 is regulated by endocytosis (26). We quantified the average number of Can1-GFP dots per cell as well as the average fluorescence intensity of Can1-GFP at the PM, in the vacuole and cytosolic dots using line intensity profiles (Fig. 3*A*). While the GFP signal intensity of the vacuole and of cytosolic dots was not affected by tricalbin deletion (Fig. 3, *C* and *D*), we show a significant increase in the number of cytosolic dots (Fig. 3*B*) and a significant decrease of signal intensity at the PM in the *tcb1*Δ*2*Δ*3*Δ mutant (Fig. 3*E*), suggesting increased endocytosis of Can1 in *tcb1*Δ*2*Δ*3*Δ strain. Canavanine is an inhibitor of protein translation that is structurally related to arginine and taken up by cells *via* Can1. We show that the *tcb1*Δ*2*Δ*3*Δ strain is resistant to canavanine (Fig. 3*F*), which is consistent with reduced Can1 expression and localization to the PM. Therefore, these results indicate that loss of tricalbins affects amino acid uptake by deregulating the expression and localization of amino acid importers such as Can1.

### Gene expression in *tcb1*Δ*2*Δ*3*Δ mimics a high-glucose environment

Transcriptional activation of amino acid synthesis depends on the transcription factor Gcn4, which is itself activated on the translational level by the kinase Gcn2 (27). Gcn2 binding to free uncharged tRNA is one prerequisite for its activation (27, 28), which is consistent with increases in tRNA synthesis (Fig. 1*B*) as well as reduced level of intracellular amino acids (Fig. 2*B*) observed in *tcb1*Δ*2*Δ*3*Δ. Moreover, Gcn2 is activated by phosphorylation as a downstream target of major regulatory pathways that respond to nutrient availability such as the TORC1 pathway (27, 29) that senses extracellular amino acids and the Snf1 pathway that senses glucose availability (30). Because we have previously shown that TORC1 activity is unaffected by deletion of tricalbin genes (12), and RNA sequencing data revealed that expression of TORC1-regulated ribosomal genes is decreased in *tcb1*Δ*2*Δ*3*Δ (91% or 116 out of 127 RPL and RPS genes are downregulated; Supplementary Dataset S1), we concluded that increases in amino acid synthesis are not caused by the activation of TORC1. Instead, we focused on potential perturbations in glucose sensing in the *tcb1*Δ*2*Δ*3*Δ mutant since it was previously reported that inactivation of the glucose sensor Snf1 induces expression of amino acid synthesis (30), especially of arginine and histidine genes that showed the strongest expression increases in the *tcb1*Δ*2*Δ*3*Δ strain (Fig. 2*A*).

**Figure 4:**
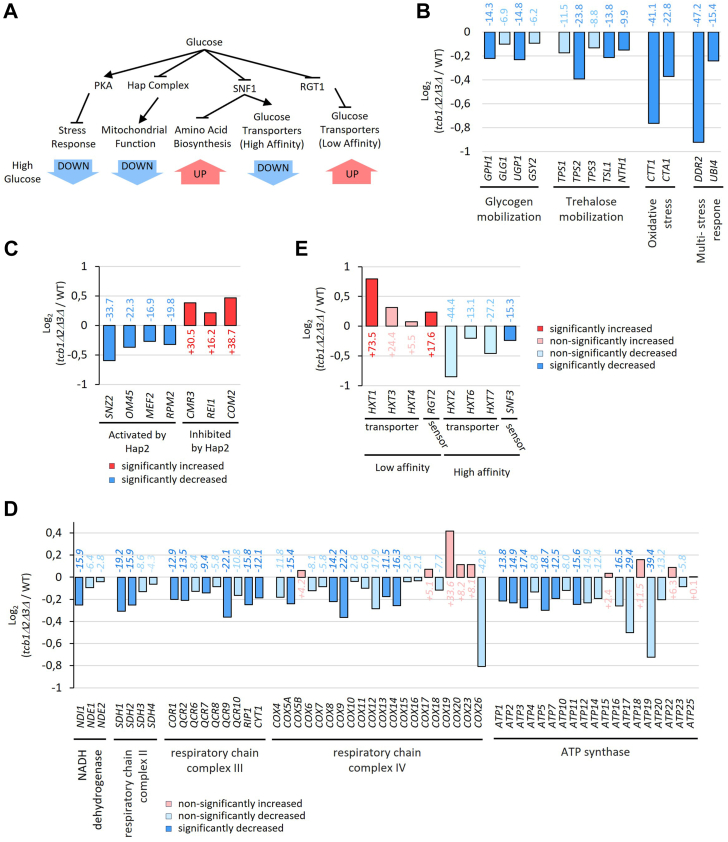
**Gene expression in *tcb1*Δ*2*Δ*3*Δ mimics a high-glucose environment.***A*, the glucose signaling network depicts regulatory pathways that mediate transcriptional changes in response to a high glucose environment. *B*, *C*, *D*, and *E*, relative expression levels between WT and *tcb1*Δ*2*Δ*3*Δ of glucose-related stress-responsive genes (*B*), Hap2-regulated genes (*C*), respiratory genes (*D*), and hexose transporter and sensor genes (*E*) depicted as Log_2_ fold change and percent value. *Dark blue* indicates significantly downregulated genes (*p* < 0.05), and *light blue* nonsignificantly downregulated genes (*p* > 0.05). Color of genes indicate significant upregulation (*p* < 0.05, *dark red*), nonsignificant upregulation (*p* > 0.05, *light red*), significant downregulation (*p* < 0.05, *dark blue*) and nonsignificant downregulation (*p* > 0.05, *light blue*).

Yeast cells sense glucose availability through various pathways and respond to a high-glucose environment with transcriptional upregulation of amino acid synthesis and low affinity glucose transport, as well as transcriptional downregulation of stress responses, mitochondrial function, and high-affinity glucose transport (Fig. 4*A*) (15, 18). If glucose is abundant, several hundred genes that are connected to various stress responses are suppressed by protein kinase A (PKA) and the transcription factors Msn2/Msn4 (31). Here, we show the suppression of major PKA-targets with a glucose-related function in *tcb1*Δ*2*Δ*3*Δ, such as glycogen and trehalose mobilization, oxidative stress, and multistress responsive genes (Fig. 4*B*). The glucose repressed heme activator protein (HAP) complex is the second major transcriptional regulator in glucose signaling that controls respiratory functions and the use of alternate carbon sources (32). Deletion of HAP complex subunit Hap2 was shown to cause a significant decrease in mitochondrial gene expression (33). We show that gene expression of *hap2*Δ is mirrored in *tcb1*Δ*2*Δ*3*Δ, although less strongly. Specifically, the four most prominently downregulated genes in *hap2*Δ are significantly reduced in *tcb1*Δ*2*Δ*3*Δ, while the most upregulated genes in *hap2*Δ are significantly increased in the *tcb1*Δ*2*Δ*3*Δ strain (Fig. 4*C*). Moreover, we show that genes of the respiratory chain and ATP synthase complexes, characteristic markers of mitochondrial function, are downregulated (Fig. 4*D*). Lastly, we examined gene expression of hexose transporters and sensors. The expression of low-affinity transporters and sensors, which are typically induced by high glucose levels (18), is increased in *tcb1*Δ*2*Δ*3*Δ, with the most substantial increase observed in *HXT1* expression (Fig. 4*E*). Conversely, glucose repressed-high affinity transporter and sensors are downregulated in *tcb1*Δ*2*Δ*3*Δ, although mostly not significantly. These changes in gene expression suggest that *tcb1*Δ*2*Δ*3*Δ may mimic the condition of cells in a more glucose-rich environment.

### Low affinity hexose transporters are enriched in the PM of *tcb1*Δ*2*Δ*3*Δ cells, whereas high-affinity hexose transporters are depleted

**Figure 5:**
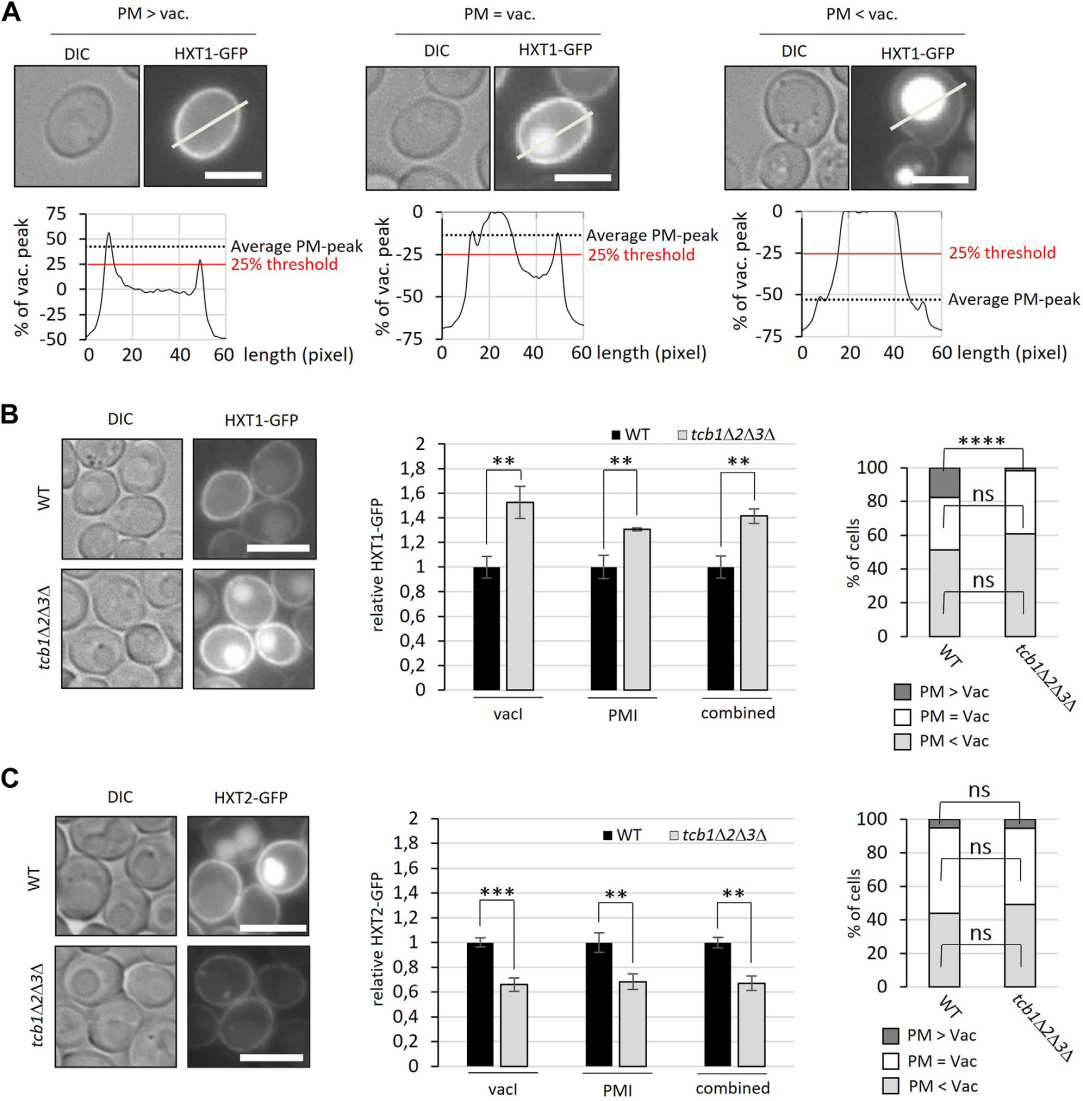
**Expression of glucose transporters is altered as if glucose is abundant in *tcb1*Δ*2*Δ*3*Δ.***A*, classification of hexose transporter localization. Single-cell fluorescence intensity was analyzed by manually positioning a line through each cell and plotting the intensity profile. Two peak intensity values for the plasma membrane and one for the vacuole were quantified, and a corresponding ratio was calculated. Cells were categorized as "PM > Vac" if the average plasma membrane peak intensity (PMI) value exceeded the peak vacuole intensity (vacI) value by 25%. Conversely, cells with a PMI value 25% lower than the vacI value were classified as "PM < vac." Cells were categorized as "PM = vac" if the PMI value did not exceed or fell below the vacI value by 25%. The image scale bar represents 5 μm. *B* and *C*, left images: WT and *tcb1*Δ*2*Δ*3*Δ cells expressing endogenously tagged *HXT1-GFP* (B) or endogenously tagged *HXT2-GFP* (*C*) were grown in YPD at 25 ˚C overnight to exponential phase and subjected to fluorescence microscopy (the image scale bar represents 5 μm). *Center* figure: PMI and vacI values were quantified for at least 100 cells and the mean PMI and vacI values normalized to WT ± SD from three independent experiments is shown. The combined value includes the mean of PMI and vacI values. Right figure: The hexose transporter localization was categorized as described in (A), and the percentage distribution as mean of three independent experiments was presented. Significance was tested using unpaired *t* test, ns>0.05, ∗*p* < 0.05, ∗∗*p* < 0.01, ∗∗∗*p* < 0.001, ∗∗∗∗*p* < 0.0001. YPD, 1% yeast extract, 2% peptone, and 2% glucose.

Hexose transporters Hxt1, Hxt2, and Hxt3 are directed for endocytic degradation upon Rsp5-dependent ubiquitination, which is induced by the accumulation of the glycolytic derivate methylglyoxal (34), and hexose transporters Hxt6 and Hxt7 have been described as a subject to glucose-induced endocytic degradation (35). Furthermore, it has been demonstrated that ER-PM contact sites facilitate endocytic membrane invagination (36). To confirm that the changes in hexose transporter gene expression translate to real changes at the protein level and to explore if tricalbin deletion affects the endocytic uptake of hexose transporters, we conducted microscopy analysis of GFP-tagged low affinity transporter Hxt1 and high-affinity transporter Hxt2 in WT and *tcb1*Δ*2*Δ*3*Δ. Fluorescence intensity profiles were obtained from individual cells, and cells were categorized based on their PM to vacuole signal ratio (Fig. 4*A*). Consistent with RNA-sequencing data that showed increased *HXT1* expression, the total fluorescence signal-intensity of Hxt1-GFP observed by microscopy significantly increased in *tcb1*Δ*2*Δ*3*Δ (Fig. 5*B*). The increase in signal intensity within the vacuole (+52%) was notably more pronounced than that at the PM (+31%). Moreover, the number of cells displaying a stronger PM signal than vacuole signal significantly decreased from 18% to 2% (Fig. 5*B*). In case of Hxt2-GFP, microscopy results showed a significant decrease in protein level in *tcb1*Δ*2*Δ*3*Δ (Fig. 5*C*), supporting the differences in *HXT2* gene expression indicated by RNAseq data. Thus, these results confirm differential expression of hexose transporters and indicate elevated endocytic uptake of Hxt1-GFP into the vacuole lumen in *tcb1*Δ*2*Δ*3*Δ.

### Differential expression of hexose transporter in *tcb1*Δ*2*Δ*3*Δ is correlated with decreased glucose uptake

**Figure 6:**
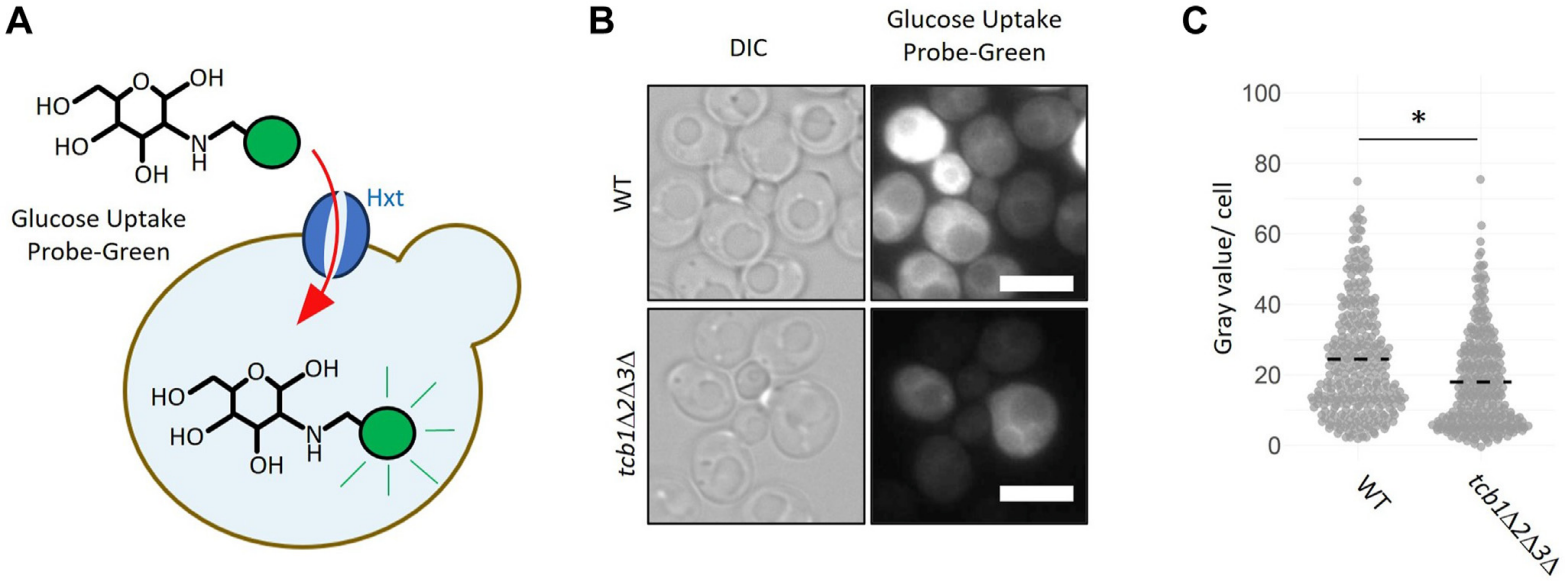
**Glucose uptake is reduced in *tcb1*Δ*2*Δ*3*Δ.***A*, a *green* fluorescent glucose analog was used to measure glucose uptake *via* hexose transporter. *B*, glucose uptake analysis of WT and *tcb1*Δ*2*Δ*3*Δ strains. Cells were cultured to exponential phase in liquid YPD and treated with a green fluorescent glucose analog (Glucose Uptake Assay *Kit-Green*; Dojindo). The uptake of the probe was measured by fluorescence microscopy. The scale bar represents 5 μm. *C*, the fluorescence intensity (*gray* value) of 100 cells from (*B*) was quantified and background adjusted. Data from three independent experiments of a total of 300 cells were presented as a beeswarm plot. Significance was tested using unpaired *t* test, ∗*p* < 0.05.

Yeast cells use multiple hexose transporters with different affinity and transport rates to adjust glucose uptake according to their needs. In a glucose rich environment, yeast cells preferably express low-affinity hexose transporter with limited glucose uptake capability (18, 37), possibly to prevent osmotic stress associated with high glucose concentrations. Conversely, low environmental glucose concentrations induce the expression of high-affinity glucose transporters to maximize uptake of the scarce resource. RNA-sequencing data indicated a shift of hexose transporter expression to the benefit of low-affinity transport in *tcb1*Δ*2*Δ*3*Δ, mimicking cells grown under glucose-rich conditions. We further showed that the changes in gene expression correlate with cellular protein levels of hexose transporters by microscopy analysis of GFP-tagged Hxt1 and Hxt2. To assess whether the reduced expression of high-affinity hexose transporters is associated with a reduced glucose transport rate, we analyzed glucose uptake using a fluorescent glucose analog (Fig. 6*A*). In the experimental setup, cells cultured to exponential phase in YPD were briefly transferred into glucose-free SC media followed by incubation with the fluorescent probe and microscopy. Our results show that the short-term glucose uptake following complete glucose depletion is reduced in *tcb1*Δ*2*Δ*3*Δ (Fig. 6, *B* and *C*). This is consistent with the fact that *tcb1*Δ*2*Δ*3*Δ expresses less high-affinity glucose transporters. Notably, this experiment does not provide clues for long-term glucose uptake under glucose-rich conditions (such as exponential growth in YPD). Because hexose transporters enable the passive uptake of glucose along a concentration gradient, long-term glucose uptake during glucose rich conditions is likely dependent on the glycolytic consumption rate. In fact, it was shown that glucose uptake in WT cells grown at high glucose concentrations is not the rate limiting step for glycolytic flux (37). Since the *tcb1*Δ*2*Δ*3*Δ strain seems to mimic gene expression of cells grown in high glucose, we next asked whether glucose consumption and metabolism is increased in the strain.

### Gene expression and metabolism of *tcb1*Δ*2*Δ*3*Δ cells are altered, resembling the response to a high-glucose environment

**Figure 7:**
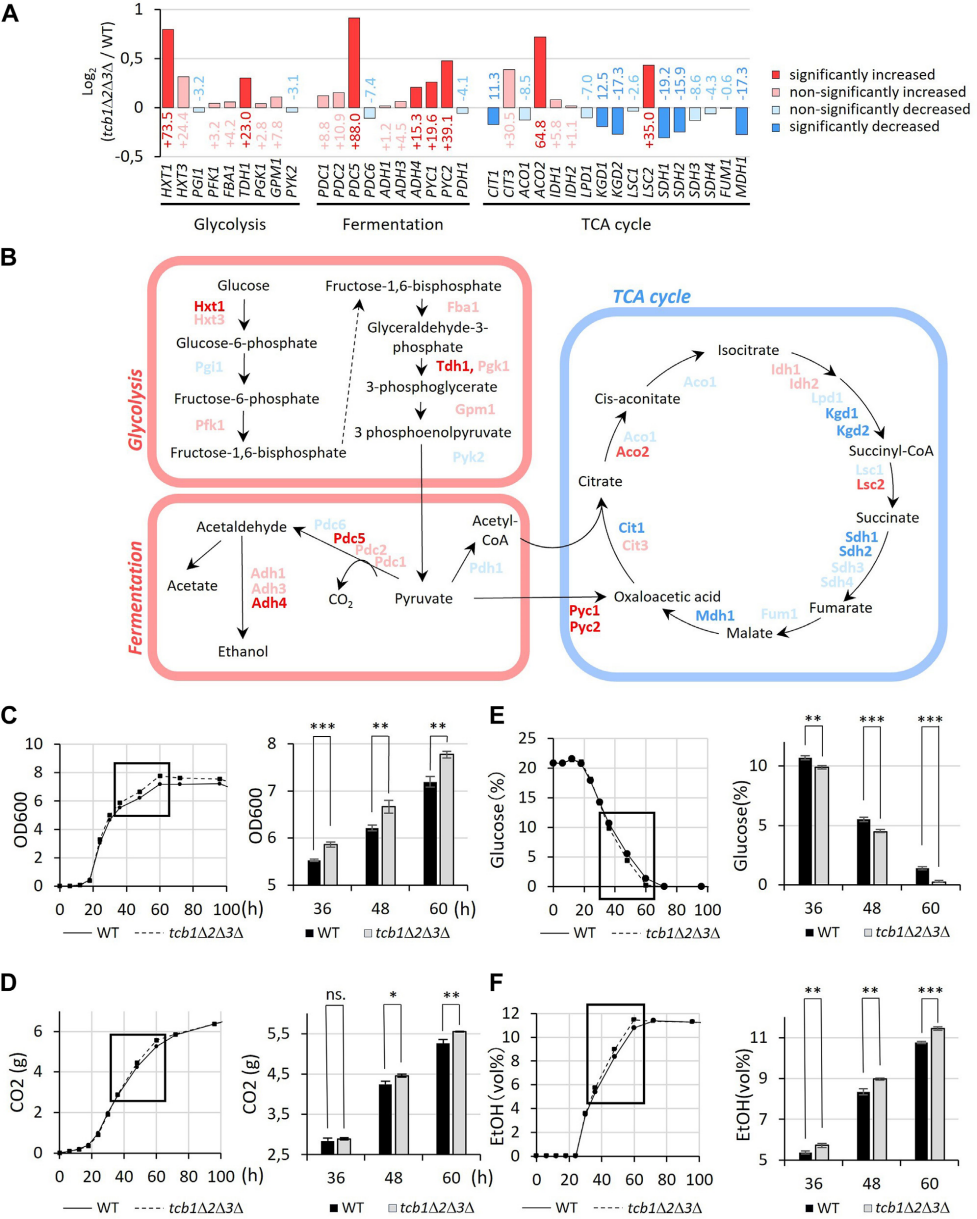
**Altered gene expression in glucose metabolism is correlated to growth, glucose consumption, CO**_**2**_**production, and ethanol production.***A*, relative expression levels between WT and *tcb1*Δ*2*Δ*3*Δ of genes involved in glycolysis, ethanol fermentation, and TCA cycle depicted as Log_2_ fold change and percent value. Color of genes indicate significant upregulation (*p* < 0.05, *dark red*), nonsignificant upregulation (*p* > 0.05, *light red*), significant downregulation (*p* < 0.05, *dark blue*) and nonsignificant downregulation (*p* > 0.05, *light blue*). Genes are arranged based on their metabolic order in glycolysis, ethanol fermentation, and TCA cycle. *B*, illustration of genes involved glycolysis, ethanol fermentation, and TCA cycle. The color of each gene corresponds to the changes in expression between WT and *tcb1*Δ*2*Δ*3*Δ and their significance, as described in (*A*). *C*, *D*, *E*, and *F*, the growth (*C*), CO_2_ production (*D*), glucose consumption (*E*), and ethanol production profiles (*F*) of WT and *tcb1*Δ*2*Δ*3*Δ strains were grown in liquid YPD medium containing 20% glucose (YPD20) and measurements taken every 6 or 12 h. Significance was tested using unpaired *t* test, ns>0.05, ∗*p* < 0.05, ∗∗*p* < 0.01, ∗∗∗*p* < 0.001. TCA, tricarboxylic acid; YPD, 1% yeast extract, 2% peptone, and 2% glucose.

We have already noted that the *tcb1*Δ*2*Δ*3*Δ strain exhibited reduced expression of mitochondrial genes. Moreover, we showed that genes involved in glycolysis, oxaloacetate synthesis, and the first steps of the citrate cycle which are connected to amino acid synthesis are upregulated in the *tcb1*Δ*2*Δ*3*Δ strain. Analysis of later steps of the citrate cycle, which are important for the regeneration of NAD+ from NADH through respiration, are downregulated in *tcb1*Δ*2*Δ*3*Δ (Fig. 7, *A* and *B*). Conversely, genes connected to fermentation, the preferred mechanism of yeast to regenerate NAD + under high glucose conditions (38), are upregulated (Fig. 7, *A* and *B*). To test whether the changes in gene expression affect cell metabolism, we analyzed differences in growth, glucose consumption, CO_2_ production, and ethanol production in WT and *tcb1*Δ*2*Δ*3*Δ. The result showed that during the late exponential growth phase, 36 to 60 h after inoculation, mass accumulation, glucose consumption, CO_2_ production, and ethanol production are significantly increased in *tcb1*Δ*2*Δ*3*Δ (Fig. 7, *C*–*F*). Thus, these findings suggest that the modified gene expression in *tcb1*Δ*2*Δ*3*Δ induces an actual shift in metabolic processes, resembling the response to a high-glucose environment.

## Discussion

MCS have been established as major hubs that control signals of various regulatory pathways (39). However, a connection of MCS to transcriptional regulation remains largely elusive. In this study, we found that deletion of the tricalbin MCS tethers causes a shift in gene expression as if cells were subjected to a high glucose environment. The apparent question that arises from this study is which step in glucose signaling is affected by tricalbins. Yeast cells respond to fluctuations in environmental glucose concentration mainly *via* three pathways: the Snf3-Rgt2 pathway, the cAMP-PKA pathway, and the Snf1-Mig1-Hxk2 pathway (17, 18). Since the signaling of all three pathways originates at the PM, it is possible that ER-PM contact sites control the early signaling steps.

**Figure 8:**
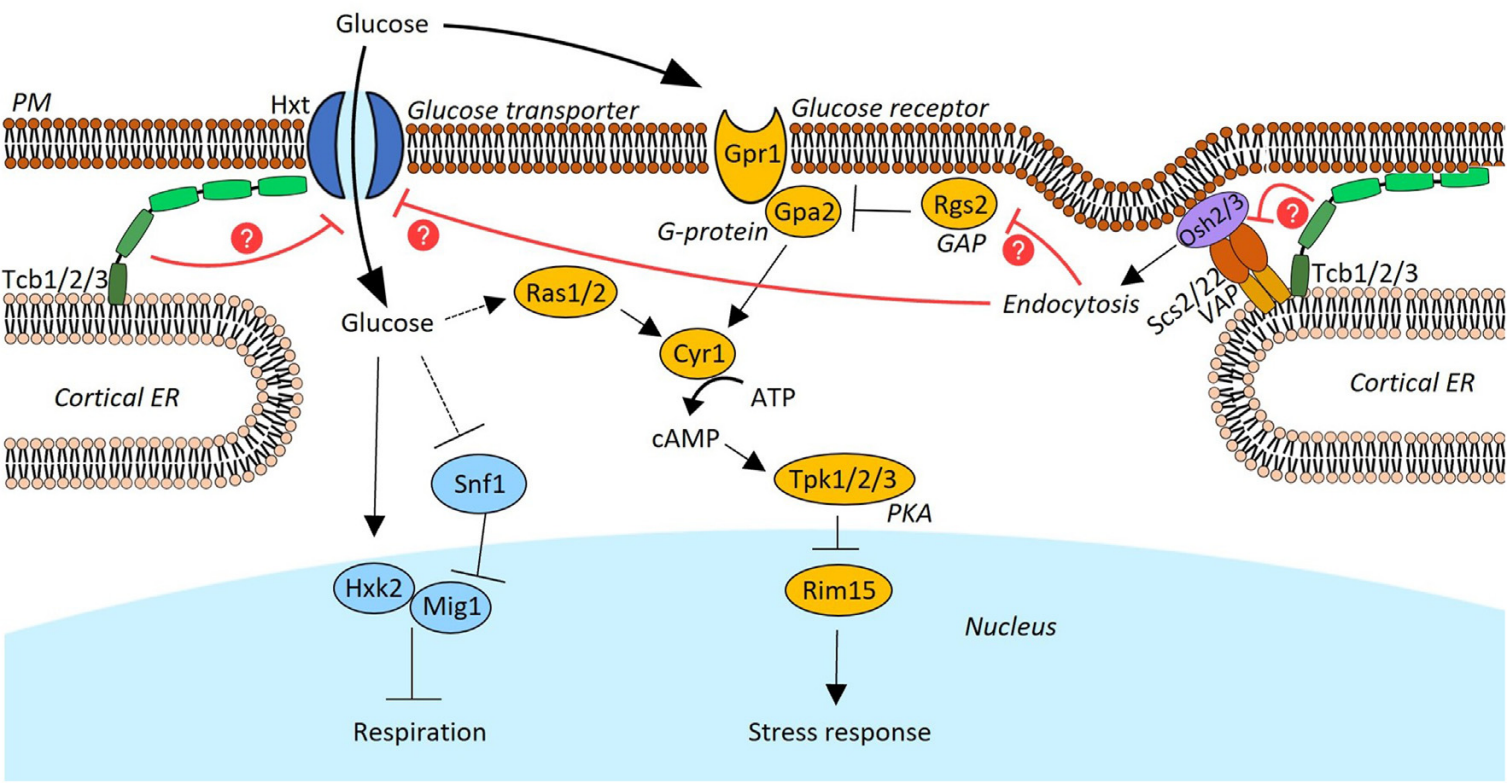
**Potential role of ER-PM MCS in glucose sensing and signaling.***Light blue*: proteins involved in Snf1-Hxk2-Mig1 signaling. *Yellow*: proteins involved in cAMP signaling. Not shown: proteins involved in Snf3/Rgt2 signaling. *Red lines* indicate potential ways how tricalbin function could suppress glucose signaling. ER, endoplasmic reticulum; MCS, membrane contact site; PM, plasma membrane.

The Snf1-Mig1-Hxk2 pathway has been suggested to sense intracellular glucose. Here, hexokinase Hxk2 serves a dual role, acting as both a catalyst for the initial reaction in glycolysis as well as a sensor of intracellular glucose and regulator of gene expression (40). In high glucose conditions, Hxk2 adopts a close conformation that promotes the formation of a complex with the transcription factor Mig1, leading to the joint repression of the SUC2 promoter (41). This promoter governs the expression of respiratory genes, which we have observed to be constitutively downregulated in the *tcb1*Δ*2*Δ*3*Δ mutant (Fig. 7, *A*–*C*). In addition, Mig1-Hxk2 complex formation is regulated by phosphorylation of Mig1 (41, 42) and Hxk2 (43) through the kinase Snf1. Although not all players in this pathway have been identified, it was suggested that the Mig1 regulation through Snf1 is also tightly linked to glucose uptake *via* PM-resident glucose transporters (44). Snf1 also regulates amino acid synthesis through the transcription factor Gcn4 and kinase Gcn2 (27) and inactivation of Snf1 caused increased arginine and histidine synthesis gene expression like in the *tcb1*Δ*2*Δ*3*Δ mutant (30, Fig. 2*A*). Consequently, the most straightforward mechanism by which tricalbins may regulate glucose signaling is by directly binding to glucose transporters at the PM in trans, thereby inactivating glucose uptake and downstream signaling (Fig. 8). Interestingly, a genome-wide *in vivo* screen for protein-protein interactions using protein-fragment complementation assay revealed that Tcb3 binds to hexose-transporter Hxt1, Hxt2, and Hxt5 (45). Therefore, deletion of tricalbins might induce a temporarily strong influx of glucose into the cell, activating glucose signaling (Fig. 8) that might in turn promote glucose consumption. Interaction of tricalbins with hexose transporters might also prevent their endocytic degradation. Tethering of the cortical ER rims to the PM through interaction of oxysterol binding protein-related proteins Osh2/3 and ER integral-membrane proteins Scs2/Scs22 was shown to facilitate membrane invagination for endocytosis (36). Tcb3, which physically interacts with hexose transporters (45), Scs2 (10) and notably the Osh family member Osh7 localized in ER-PM MCS (45, 46), might negatively regulate endocytosis. Consequently, deletion of tricalbins might facilitate the endocytic uptake of PM-resident proteins, which we have observed in case of Can1 (Fig. 3*B*) and Hxt1 (Fig. 5*B*).

The cAMP-PKA pathway represents the second major glucose signaling pathway and senses both intra and extracellular glucose (18) (Fig. 8). In this pathway, the adenylate cyclase Cyr1 is activated in response to glucose abundance by two distinct mechanisms to generate the second messenger cAMP. Subsequently, cAMP suppresses genes involved in postdiauxic shift and stress responses, a repression that we have constitutively observed in the tricalbin mutant (Fig. 4*B*). Activation of Cyr1 is induced either through the PM-resident GTPase Ras1 that responds to intracellular glucose (47, 48, 49), or by the PM-resident G protein-coupled receptor Gpr1, which binds extracellular glucose and activates Cyr1 through its G-protein Gpa2 (49, 50). Rgs2, the GTPase activating protein (GAP) of Gpa2, has been described as a negative regulator of glucose-induced cAMP signaling (51). Similar to the discussed function of ER-PM contacts in the endocytosis of yeast, a recent study in plants indicated a function of Osh-homolog ORP2A and Scs2 homolog VAP27-1 in the glucose induced endocytic degradation of the Rgs2 homolog RGS1 (52). If Rgs2 is targeted for endocytic degradation upon glucose stimulation likewise in yeast, deletion of tricalbins might promote endocytosis, Rgs2 degradation, and relief Rgs2-mediated inhibition on cAMP-PKA signaling (Fig. 8). Therefore, ER-PM contacts might regulate the cAMP-PKA pathway either through controlling glucose uptake or by affecting G-protein signaling at the PM.

Lastly, the Snf3/Rgt2 pathway senses extracellular glucose through the PM-resident sensor proteins Snf3 and Rgt2, which activate a kinase signaling cascade and induce the expression of high- or low-affinity glucose transporters according to need (53, 54). Our results showed that gene expression of hexose transporters and sensors is significantly changed in *tcb1*Δ*2*Δ*3*Δ to the benefit of low affinity hexose transport (Figs. 4*E* and 8). Snf3 and Rgt2-signaling depends on their large cytosolic domains that interact with the downstream factor Yck1 at the PM (55, 56). Thus, ER-PM contacts could also be involved in this process.

Tethering proteins are known to relocalize to different contact sites as downstream effectors of various stress response mechanisms (2, 20). Regarding tricalbins, we have previously reported that Tcb3 localizes to ER-Golgi contacts during ER-stress to facilitate nonvesicular ceramide transport (11). In addition, we have shown that tricalbin deletion causes accumulation of the sphingolipid precursor phytosphingosine and phytosphingosine-induced fragmentation of the vacuole membrane (12) as well as accumulation of lipid droplets (11). Our results show that the loss of *VPS16*, a subunit of the HOPS and CORVET complexes, promotes the uptake of glucose (Fig. S2), suggesting that the reduced glucose uptake observed in the *tcb1*Δ*2*Δ*3*Δ is not due to fragmented vacuoles. This raises the possibility that the perturbed glucose signaling observed in the *tcb1*Δ*2*Δ*3*Δ mutant may be a result of the disruption of contact sites other than the cortical ER. The nucleus vacuole junction, for example, expands upon glucose exhaustion through recruitment of the tethering protein Snd3 (21) and mediates several responses to low glucose level including adjusting mevalonate metabolism, lipid droplet synthesis, and vacuolar membrane microdomain formation (22, 57, 58). Moreover, the tethering of contact sites between vacuole and mitochondria (vCLAMP) and ER mitochondria (ERMES) is oppositely regulated by glucose, which promotes vCLAMP and inhibits ERMES formation (59, 60). Off note, studies on mice indicated that contacts of the ER with mitochondria facilitate the interaction of glycolytic enzymes and promote the switch from fatty acids to glucose-derived pyruvate as a main source for respiration (61).

Why is glucose signaling regulated through MCSs? Under real-world conditions, yeast cells often find themselves subjected to a combination of different environmental stresses. Contact sites between the ER and PM have been shown to regulate responses to heat stress, cell wall integrity, endocytosis and exocytosis, osmotic stress, and perturbation of lipid and calcium homeostasis (1). Maintaining such a variety of functions with a very limited number of tethering proteins is only possible due to the often multifunctional and inducible nature of tethering proteins (which are themselves targets of regulation). For instance, the mammalian ER-PM tethering protein TMEM24 is essential for sustaining intracellular Ca^2+^ oscillations that trigger bursts of insulin granule release in response to high glucose levels (62). In this process, TMEM24 associates and dissociates with the PM in cycles, facilitating Ca^2+-^induced phosphatidylinositol transport from the ER to the PM while connected. This way, TMEM24 connects the three signaling pathways for glucose, calcium, and phosphatidylinositol-4-phosphate. Similarly, phospholipid transport at ER-PM contacts mediated by tricalbins is increased when calcium is present (63). Moreover, the direct homolog of tricalbins, mammalian E-Syt1, seems to play an antagonistic role to TEMEM24 as it associates with the PM upon Ca^2+^ stimulation when TEMEM24 is dissociated (64, 65, 66). Therefore, tricalbin-tethered ER-PM contact sites might serve as an interface that balances the early glucose signaling response with PM lipid homeostasis and Ca^2+^ signaling. As of this multifunctional nature of tethers, one would expect that next to glucose signaling, other transcriptionally regulated signaling pathways are affected by tricalbin deletion too. In fact, we found that the strongest changes in gene expression concerned genes related to mating. Receptors for a and alpha pheromones, Ste2/3, belong to the highest differentially expressed genes (Fig. 1*A*) and are the only other two G-proteins that have been described in yeast next to the glucose sensor Gpa2 (67), possibly suggesting a connection of tethers to G-protein signaling. Recently Quon *et al.* investigated the changes in gene expression upon deletion of all ER-PM tethering proteins (Δ*-s-tether*) and upon deletion of ER-PM MCS resident Osh proteins (24). The study reported a joint function of ER-PM MCS and Osh-proteins in environmental stress response, as both deletion of tethers and Osh proteins caused upregulation of induced environmental stress response, in particular the unfolded protein response, the high-osmolarity glycerol pathway and to some degree the heat shock response. Here, we found that if tricalbins are deleted alone, stress genes related to glucose limitation are repressed (Fig. 4*B*). Many heat shock proteins, such as the bi-chaperone system, Hsp12 or Hsp70 (yeast Ssa1-Ssa4) mediate responses to ethanol stress and are induced during diauxic shift when glucose becomes depleted (68, 69, 70). Accordingly, we found that heat shock genes are strongly repressed in *tcb1*Δ*2*Δ*3*Δ (Fig. S3 and Supplementary Dataset S2). The osmotic stress response partially induces the same set of heat shock genes but furthermore glycerol synthesis genes (69, 70, 71). Interestingly, besides downregulation of heat shock genes, expression of the osmostress-regulated isoform of the glycerol synthase, Gpd1 (72, 73), was not affected by tricalbin-deletion whereas expression of the Gpd2 isoform that produces glycerol to achieve redox balance under anaerobic and high-glucose growth conditions was significantly increased (Fig. S3 and Supplementary Dataset S3) (74, 75, 76). Analysis of unfolded protein response genes in *tcb1*Δ*2*Δ*3*Δ showed overall upregulation similarly but less strongly to Δ*-s-tether* (Fig. S3, Supplementary Dataset S4) (24).

To summarize, we have found that deletion of the tricalbin tethering proteins induces a shift in gene expression and metabolism mimicking the cellular response to a high-glucose environment. It will be of particular interest to understand mechanistically how tricalbins are involved in glucose sensing and signaling processes.

## Experimental procedures

### Yeast strains and media

Yeast strains used in this study are listed in Table S1. Yeast cultivations, genetic manipulation, and strain construction were carried out as described previously (77). The *tcb1*Δ*2*Δ*3*Δ strain and GFP-tagged WT strains were generated by PCR based one step gene replacement/attachment and confirmed by colony PCR using primer binding outside of the targeted gene. GFP-tagged *tcb1*Δ*2*Δ*3*Δ strains were generated by mating of WT *HXT1-GFP* and WT *HXT2-GFP* with *tcb1*Δ*2*Δ*3*Δ.

### RNA sequencing

RNA was extracted from WT and *tcb1*Δ*2*Δ*3*Δ cells cultured in YPD (liquid medium overnight at 25 °C to *A*_600_ = 0.6 in triplicates using the RNeasy Mini Kit (Qiagen). The recovered RNA samples were contracted to BGI Group (https://www.bgi.com/jp/home) for analysis and data processing. The complete and raw RNA sequencing data can be found in Supplementary Dataset S5.

### Total amino acid analysis

Amino acids were extracted from whole cells cultured overnight at 25 °C in YPD liquid medium to *A*_600_ = 0.6 and measured by an automatic amino acid analyzer (JEOL JLC-500/V) as described previously (78).

### Canavanine treatment

WT and *tcb1*Δ*2*Δ*3*Δ strains were grown in liquid synthetic defined medium (SD; 2% glucose, 0.5% ammonium sulfate, 0.17% yeast nitrogen base; 80 mg/L amino acids/base Ade, Ura, Leu, Lys, His, and Trp) at 25 °C overnight to exponential phase. Fivefold serial dilutions of cells corresponding to an initial *A*_600_ = 1 were spotted on SD plates containing canavanine in different concentrations (0, 1, and 8 μg/ml) and were incubated at 25 °C for 3 days.

### Fluorescent microscopy of GFP-tagged proteins

WT *HXT1-GFP*, WT *HXT2-GFP*, *tcb1*Δ*2*Δ*3*Δ *HXT1-GFP, tcb1*Δ*2*Δ*3*Δ *HXT2-GFP,* WT *CAN1-GFP, and tcb1*Δ*2*Δ*3*Δ *CAN1-GFP* strains were grown in SD medium at 25 °C overnight to exponential growth phase and imaged by differential interference contrast and fluorescence microscopy. Images were captured using an Olympus BX51 microscope with a CCD camera (Retiga, R3), a 100 × objective (Olympus, UPlanApo) and a filter permissive for excitation wavelengths between 460 and 480 nm and detection wavelength between 495 and 540 nm, thus suitable to GFP. An exposure time of 1s was used for cell expressing *HXT1-GFP*, 2s for cells expressing *HXT2-GFP*, and 3s for cells expressing *CAN1-GFP.* Pictures were processed as laid out in Figures 3*A* and 6*A* using ImageJ software (https://imagej.net/ij/).

### Glucose uptake

WT, *tcb1*Δ*2*Δ*3*Δ (Fig. 6) as well as BY WT and BY *vps16Δ* (Fig. S2) strains were grown to exponential phase in liquid YPD, washed with synthetic complete media without glucose (SC-Glucose; 0.17% yeast nitrogen base, 0.5% ammonium sulfate, and 1.3 g/l dropout powder) and incubated for another 15 min in SC-Glucose. Cells were treated with 500X Uptake Probe from Glucose Uptake Assay Kit-Green (Dojindo) for 30 min, washed with WI solution (Dojindo) and observed by fluorescence microscopy. Images were captured using an Olympus BX51 microscope with a CCD camera (Retiga, R3), a 100 × objective (Olympus, UPlanApo)). All pictures were taken using the appropriate filter for green fluorescence (460–480 nm excitation and 495–540 nm emission) and using 200 ms exposure time for WT *versus tcb1*Δ*2*Δ*3*Δ and 800 ms exposure time for WT *versus vps16Δ*. Fluorescence intensity was measured by drawing region of interests around 100 single cells in ImageJ, assessing the gray value, and subtracting the gray value of the background.

### Growth and metabolic profiles

WT and *tcb1*Δ*2*Δ*3*Δ strains were grown in liquid YPD medium at 30 °C containing 20% glucose (YPD20: 1% yeast extract, 2% peptone, and 20% glucose) and measurements for growth and metabolic profiles were taken after 0, 6, 12, 18, 24, 30, 36, 48, 60, 72, 96, 120, 200, and 260 h. *A*_600_ values were measured for growth profiles. For ethanol and glucose concentrations, cells grown in YPD20 medium were centrifuged and the supernatant was filtered using Captiva Econofilter 0.45 μm (Agilent Technologies, Inc). Ethanol concentration in the filtrate was determined using an 8890 Gas Chromatograph (GC) System (Agilent Technologies, Inc) with n-propanol as an internal standard. Glucose concentration was measured using an ADAMS Glucose GA-1153 analyzer (Arkray Inc) according to the manufacturer’s protocol. For CO_2_ production, total culture weight was measured, and the decrease in weight at each time point calculated as described previously (79).

### Statistical analysis

Tukey–Kramer multiple comparison test was performed for glucose uptake experiments, combining data from three independent experiments. For other experiments, statistical analysis was performed using Student’s *t* test calculated from three independent experiments. For all tests, *p* values are classified: ns not significant; ∗, *p* < 0.05; ∗∗, *p* < 0.01 and ∗∗∗, *p* < 0.001. The mean ± sd for three independent experiments is shown.

## Data availability

All data generated or analyzed during this study are contained within the article.

## Supporting information

This article contains supporting information (24).

## Conflict of interest

The authors declare that they have no conflicts of interest with the contents of this article.

## Supporting information

Supporting information Figures S1 to S3

Supporting information Datasets S1 to S5
