# Supplementary material for: The tricalbin family of membrane contact site tethers is involved in the transcriptional responses of Saccharomyces cerevisiae to glucose
Source: J Biol Chem. 2024 Aug 10;300(9):107665. doi: 10.1016/j.jbc.2024.107665 (PMC11408865; doi:10.1016/j.jbc.2024.107665)
Supplement: Supporting information Figures S1 to S3 [file mmc2.pptx]

## Slide 1
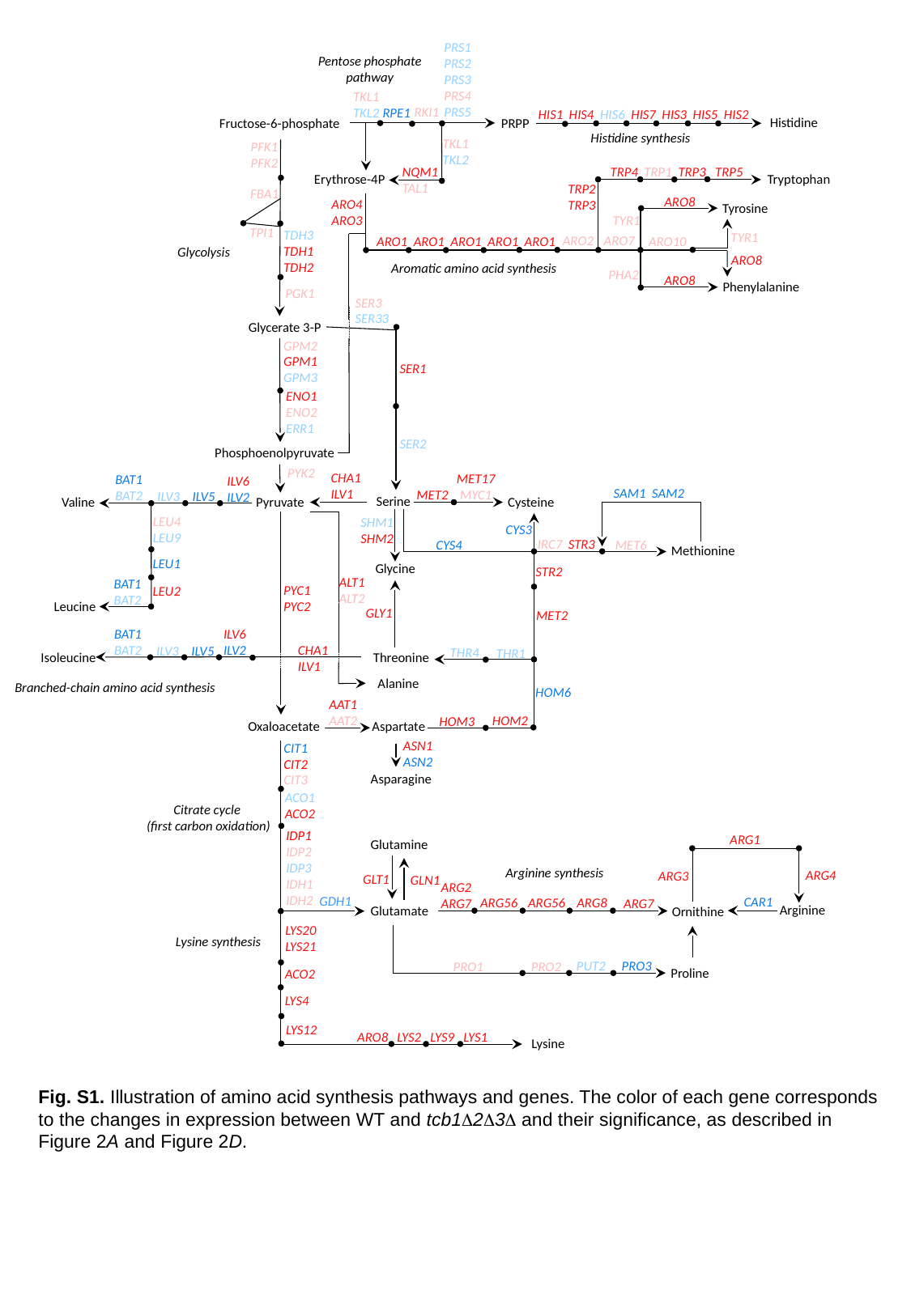

PRS1 PRS2 PRS3 PRS4 PRS5
Pentose phosphate pathway
TKL1
TKL2
RKI1
RPE1
HIS1 HIS4 HIS6 HIS7 HIS3 HIS5 HIS2
Histidine
Fructose-6-phosphate
PRPP
Histidine synthesis
TKL1
TKL2
PFK1
PFK2
NQM1 TAL1
TRP4 TRP1 TRP3 TRP5
Erythrose-4P
Tryptophan
TRP2
TRP3
FBA1
ARO8
ARO4
ARO3
Tyrosine
TYR1
TPI1
TDH3 TDH1 TDH2
TYR1
ARO7
ARO2
ARO1 ARO1 ARO1 ARO1 ARO1
ARO10
Glycolysis
ARO8
Aromatic amino acid synthesis
PHA2
ARO8
Phenylalanine
PGK1
SER3 SER33
Glycerate 3-P
GPM2 GPM1 GPM3
SER1
ENO1 ENO2 ERR1
SER2
Phosphoenolpyruvate
PYK2
CHA1 ILV1
MET17 MYC1
BAT1 BAT2
ILV6 ILV2
SAM1 SAM2
MET2
ILV3
ILV5
Serine
Valine
Cysteine
Pyruvate
LEU4 LEU9
SHM1 SHM2
CYS3
IRC7 STR3
CYS4
MET6
Methionine
LEU1
Glycine
STR2
ALT1 ALT2
BAT1 BAT2
PYC1 PYC2
LEU2
Leucine
GLY1
MET2
BAT1 BAT2
ILV6 ILV2
CHA1 ILV1
ILV3
ILV5
THR4
THR1
Threonine
Isoleucine
Alanine
Branched-chain amino acid synthesis
HOM6
AAT1 AAT2
HOM2
HOM3
Oxaloacetate
Aspartate
ASN1 ASN2
CIT1 CIT2 CIT3
Asparagine
ACO1 ACO2
Citrate cycle
 (first carbon oxidation)
IDP1 IDP2 IDP3 IDH1 IDH2
ARG1
Glutamine
ARG4
ARG3
GLT1
GLN1
ARG2 ARG7
GDH1
CAR1
ARG56
ARG56
ARG8
ARG7
Arginine
Glutamate
Ornithine
LYS20 LYS21
PRO3
PUT2
PRO1
PRO2
Proline
ACO2
LYS4
LYS12
ARO8 LYS2 LYS9 LYS1
Lysine
Arginine synthesis
Lysine synthesis
Fig. S1. Illustration of amino acid synthesis pathways and genes. The color of each gene corresponds to the changes in expression between WT and tcb1D2D3D and their significance, as described in Figure 2A and Figure 2D.

## Slide 2
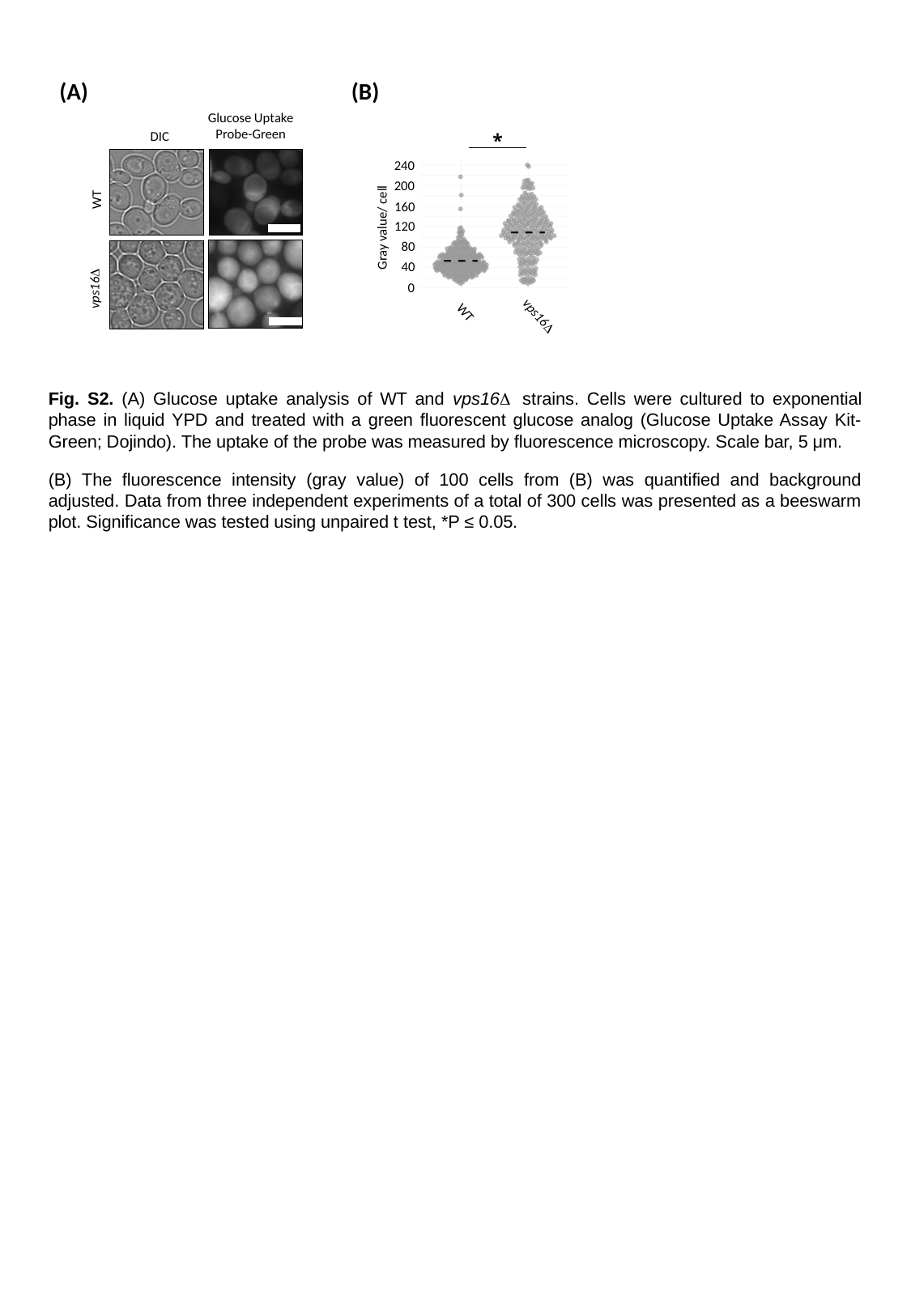

(A)
(B)
Glucose Uptake Probe-Green
DIC
*
240
200
WT
160
120
Gray value/ cell
80
40
0
vps16D
WT
vps16D
Fig. S2. (A) Glucose uptake analysis of WT and vps16D strains. Cells were cultured to exponential phase in liquid YPD and treated with a green fluorescent glucose analog (Glucose Uptake Assay Kit-Green; Dojindo). The uptake of the probe was measured by fluorescence microscopy. Scale bar, 5 μm.
(B) The fluorescence intensity (gray value) of 100 cells from (B) was quantified and background adjusted. Data from three independent experiments of a total of 300 cells was presented as a beeswarm plot. Significance was tested using unpaired t test, *P ≤ 0.05.

## Slide 3
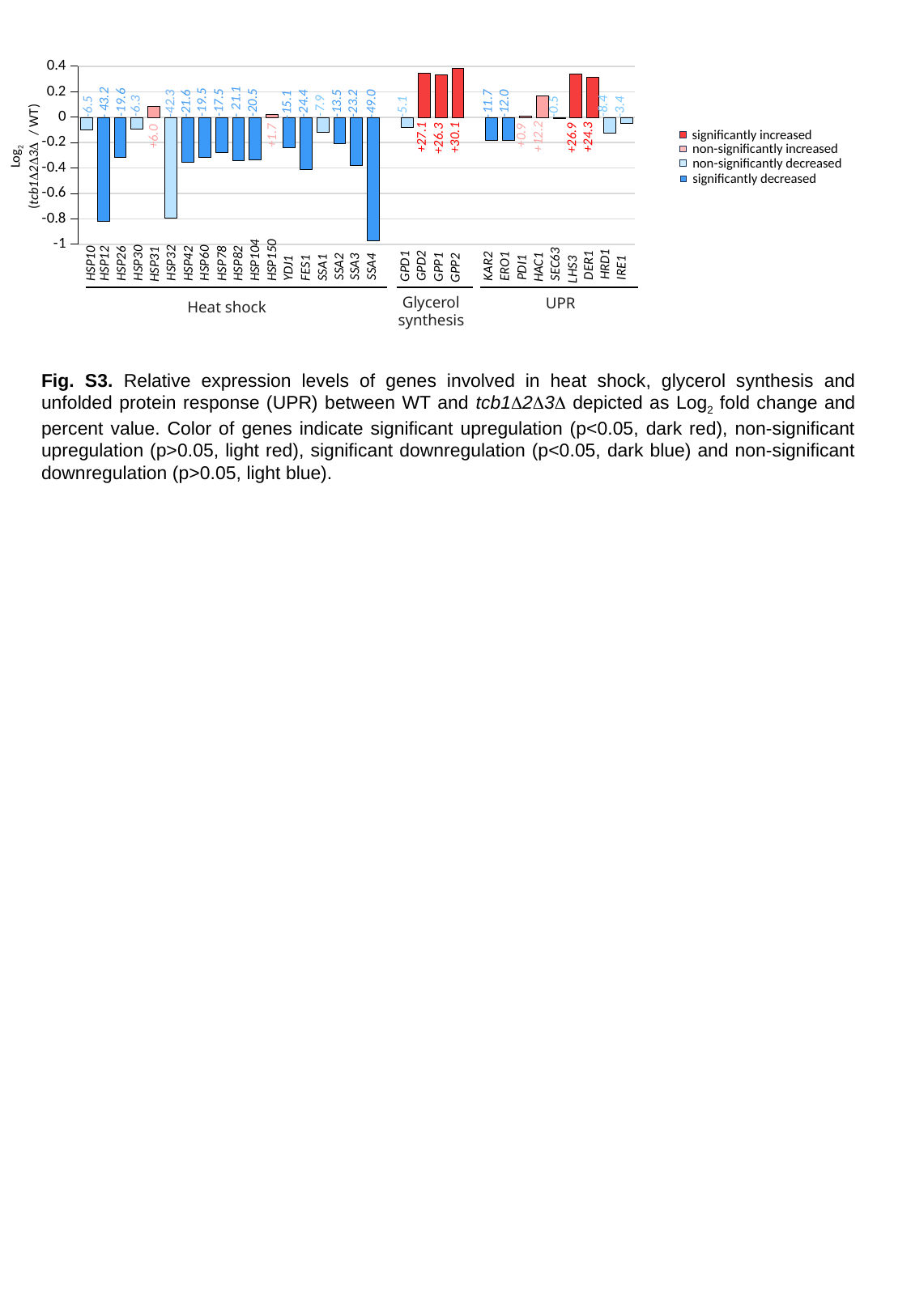

### Chart
| Category | 'HSP10' |
|---|---|
| GPH1 | -0.096652181 |
| GLG1 | -0.814916845 |
| UGP1 | -0.315505839 |
| GSY2 | -0.093429583 |
| | 0.083764392 |
| TPS1 | -0.793936834 |
| TPS2 | -0.35053319 |
| TPS3 | -0.312929684 |
| TSL1 | -0.277537439 |
| NTH1 | -0.342740317 |
| | -0.33097444 |
| CTT1 | 0.024557981 |
| 'CTA1' | -0.23625756 |
| | -0.409866078 |
| DDR2 | -0.118150638 |
| UBI4 | -0.209491942 |- 21.1
-19.5
-19.6
-20.5
-17.5
- 43.2
-23.2
-24.4
-42.3
-21.6
-12.0
-13.5
-49.0
-11.7
-15.1
-7.9
-6.3
-8.4
-0.5
-6.5
-5.1
-3.4
significantly increased
+1.7
+6.0
+24.3
+27.1
+30.1
+0.9
+26.3
+12.2
Log2
(tcb1D2D3D / WT)
non-significantly increased
+26.9
non-significantly decreased
significantly decreased
HSP150
HSP104
HSP30
HSP32
HSP60
HSP82
HSP78
HSP10
HSP12
HSP26
HSP42
HRD1
SEC63
HSP31
GPD2
ERO1
DER1
SSA4
GPD1
KAR2
SSA3
SSA2
HAC1
GPP1
IRE1
SSA1
FES1
GPP2
YDJ1
PDI1
LHS3
Glycerol synthesis
UPR
Heat shock
Fig. S3. Relative expression levels of genes involved in heat shock, glycerol synthesis and unfolded protein response (UPR) between WT and tcb1D2D3D depicted as Log2 fold change and percent value. Color of genes indicate significant upregulation (p<0.05, dark red), non-significant upregulation (p>0.05, light red), significant downregulation (p<0.05, dark blue) and non-significant downregulation (p>0.05, light blue).
