# Supplementary material for: The tricalbin family of membrane contact site tethers is involved in the transcriptional responses of Saccharomyces cerevisiae to glucose
Source: J Biol Chem. 2024 Aug 10;300(9):107665. doi: 10.1016/j.jbc.2024.107665 (PMC11408865; doi:10.1016/j.jbc.2024.107665)
Supplement: Supporting information Datasets S1 to S5 [file mmc3.docx]

**Supporting Information for**

The tricalbin family of membrane contact site tethers is involved in the transcriptional responses of *S. cerevisiae* to glucose

Philipp Schlarmann^1^, Keiko Sakuragi^1^, Atsuko Ikeda^1^, Yujia Yang^1^, Saku Sasaki^1^, Kazuki Hanaoka^1^, Misako Araki^1^, Tomoko Shibata^2^, Muneyoshi Kanai^2^, Kouichi Funato^1^*

^1^Graduate School of Integrated Sciences for Life, Hiroshima University, Kagamiyama 1-4-4, Higashi-Hiroshima 739-8528, Japan.

^2^National Research Institute of Brewing, 3-7-1 Kagamiyama, Higashi, Hiroshima 739-0046, Japan.

* Corresponding author; Kouichi Funato

**Email:**  kfunato@hiroshima-u.ac.jp

**Description to Supplemental Figures S1 and S2:**

Fig. S1. Illustration of amino acid synthesis pathways and genes. The color of each gene corresponds to the changes in expression between WT and *tcb1*Δ*2*Δ*3*Δ and their significance, as described in Figure 2*A* and Figure 2*D*.

**Fig. S2.** (A) Glucose uptake analysis of WT and *vps16*Δ strains. Cells were cultured to exponential phase in liquid YPD and treated with a green fluorescent glucose analog (Glucose Uptake Assay Kit-Green; Dojindo). The uptake of the probe was measured by fluorescence microscopy. Scale bar, 5 μm.

(B) The fluorescence intensity (gray value) of 100 cells from (B) was quantified and background adjusted. Data from three independent experiments of a total of 300 cells was presented as a beeswarm plot. Tukey’s test using combined data from three independent experiments: ***P ≤ 0.001.

Fig. S3. Relative expression levels of genes involved in heat shock, glycerol synthesis and unfolded protein response (UPR) between WT and *tcb1*Δ*2*Δ*3*Δ depicted as Log_2_ fold change and percent value. Color of genes indicate significant upregulation (p<0.05, dark red), non-significant upregulation (p>0.05, light red), significant downregulation (p<0.05, dark blue) and non-significant downregulation (p>0.05, light blue).

**Supporting Tables**

| Strain No | Stain name | Genotype | Source |
| --- | --- | --- | --- |
| FKY5663 | WT | MAT**alpha** *ura3 leu2 his3 trp1 bar1 lys2* | This study |
| FKY5665 | *tcb1Δ2Δ3Δ* | MAT**a** *ura3 trp1 bar1 lys2 tcb1Δ::KanMX tcb2Δ::HIS3 tcb3Δ::LEU2* | This study |
| FKY6175 | *WT*  *HXT1-GFP* | MAT**alpha** *ura3 leu2 his3 trp1 bar1 lys2 HXT1-GFP::TRP1* | This study |
| FKY6176 | *WT*  *HXT2-GFP* | MAT**alpha** *ura3 leu2 his3 trp1 bar1 lys2 HXT2-GFP::TRP1* | This study |
| FKY6384 | *tcb1Δ2Δ3Δ HXT1-GFP* | MAT**alpha** *ura3 trp1 his3 bar1 lys2 tcb1Δ::KanMX tcb2Δ::HIS3 tcb3Δ::LEU2 HXT1-GFP::TRP1* | This study |
| FKY6390 | *tcb1Δ2Δ3Δ HXT2-GFP* | MAT**alpha** *ura3 trp1 his3 bar1 lys2 tcb1Δ::KanMX tcb2Δ::HIS3 tcb3Δ::LEU2 HXT2-GFP::TRP1* | This study |
| FKY6911 | *WT CAN1-GFP* | MAT**alpha** *ura3 leu2 his3 trp1 bar1 lys2 CAN1GFP-URA* | This study |
| FKY6968 | *tcb1Δ2Δ3Δ CAN1-GFP* | MAT**alpha** *ura3 trp1 bar1 lys2 tcb1Δ::KanMX tcb2Δ::HIS3 tcb3Δ::LEU2 CAN1GFP-URA* | This study |
| FKY1772 | *WT (BY4742)* | MAT**alpha** *his3 leu2 lys2 ura3* | This study |
| Knockout collection  (YKC123-66) | *vps16Δ*  *(BY4742)* | MAT**alpha** *his3 leu2 lys2 ura3 vps16Δ::KanMX* | This study |

Table S1. Yeast strains used in this study.

| Name | Log_2_ fold change | Log_10_  q value | Function |  | Name | Log_2_ fold change | Log_10_  q value | Function |
| --- | --- | --- | --- | --- | --- | --- | --- | --- |
| *YHR054C* | 20,58 | 5,33 | Unknown |  | *SHR5* | 1,30 | 21,08 | Lipid metab. (Palmitolyation) |
| *HMRA1* | 7,06 | 2,91 | Mating |  | *YGK3* | 1,27 | 8,55 | Protein degradation (regulation) |
| *ASG7* | 6,51 | 25,48 | Mating |  | *LEU2* | 1,26 | 36,56 | Aminoacid synthesis |
| *AGA2* | 6,37 | 2,29 | Mating |  | *RIB4* | 1,26 | 52,85 | Other (Lumazine synthesis) |
| *YMR051C* | 6,19 | 3,61 | Retrotransposon |  | *BDS1* | 1,25 | 22,59 | Other (sulfatase) |
| *STE2* | 5,81 | 25,29 | Mating |  | *AQR1* | 1,22 | 8,57 | Other (amino acid export) |
| *YHL009W-A* | 5,23 | 1,92 | Retrotransposon |  | *YGL117W* | 1,22 | 17,93 | Unknown |
| *ARG3* | 4,59 | 24,66 | Aminoacid synthesis |  | *OPT2* | 1,20 | 5,98 | Peroxisome |
| *SNO1* | 4,14 | 4,18 | Unknown (Pyridoxin) |  | *ZPS1* | 1,19 | 3,06 | Unknown |
| *ARG1* | 4,01 | 46,62 | Aminoacid synthesis |  | *YOL107W* | 1,19 | 15,53 | Unknown |
| *BAR1* | 3,86 | 129 | Mating |  | *YPS3* | 1,18 | 2,4 | Cell wall |
| *HIS3* | 3,85 | 250,84 | Aminoacid synthesis |  | *YJL213W* | 1,17 | 12,76 | Unknown (Ribosome) |
| *STE6* | 3,22 | 183,76 | Mating |  | *YGR038CB* | 1,17 | 6,26 | Retrotransposon |
| *ARG8* | 2,99 | 27,63 | Aminoacid synthesis |  | *PPM2* | 1,17 | 3,67 | tRNA (methyltransferase) |
| *ARG5,6* | 2,66 | 30,54 | Aminoacid synthesis |  | *CSS1* | 1,16 | 1,67 | Unknown |
| *CPA1* | 2,51 | 32,85 | Aminoacid synthesis |  | *PFK27* | 1,15 | 46,12 | Glucose metabolism |
| *YHR214W* | 2,15 | 4,43 | Unknown |  | *PSF3* | 1,15 | 20,76 | Other (DNA amplification) |
| *YAR068W* | 1,99 | 6,12 | Unknown |  | *AMS1* | 1,15 | 1,77 | Glucose metabolism |
| *TIR1* | 1,84 | 1,31 | Cell wall |  | *RRP40* | 1,14 | 31,31 | Transcription |
| *HIS4* | 1,82 | 75,43 | Aminoacid synthesis |  | *TMT1* | 1,13 | 26,41 | Aminoacid synthesis |
| *YKL071W* | 1,77 | 2,05 | Other (oxidoreductase) |  | *PEX11* | 1,12 | 14,82 | Peroxisome |
| *MCH4* | 1,62 | 49,52 | Mating |  | *PTH4* | 1,12 | 7,82 | tRNA (peptidyl-tRNA hydrolase) |
| *CSS3* | 1,60 | 16,67 | Unknown |  | *SKM1* | 1,11 | 36,23 | Protein kinase |
| *CPA2* | 1,57 | 52,81 | Aminoacid synthesis |  | *WSC3* | 1,10 | 16,67 | Cell wall |
| *IMA2* | 1,56 | 11,06 | Glucose metabolism |  | *RTC1* | 1,10 | 39,26 | Other (TORC) |
| *YER160C* | 1,55 | 50,25 | Retrotransposon |  | *TRM13* | 1,08 | 26,28 | tRNA (methyltransferase) |
| *YOR343C* | 1,49 | 1,44 | Unknown |  | *HRT1* | 1,08 | 14,31 | Protein degradation (ubiquitinylation) |
| *PIR3* | 1,47 | 2,79 | Cell wall |  | *MED7* | 1,08 | 35,92 | Transcription |
| *PRM2* | 1,43 | 2,19 | Mating |  | *TIR3* | 1,08 | 2,12 | Cell wall |
| *GAS4* | 1,43 | 1,83 | Cell wall |  | *DCP1* | 1,05 | 29,92 | Other (DNA amplification) |
| *GCV1* | 1,43 | 2,82 | Glycine catabolism |  | *PDR11* | 1,05 | 9,85 | Other (sterol transporter) |
| *YDR461C-A* | 1,41 | 10,68 | Unknown |  | *TRM11* | 1,03 | 17,74 | tRNA (methyltransferase) |
| *ARG7* | 1,41 | 52,55 | Aminoacid synthesis |  | *MDH2* | 1,03 | 34,69 | Glucose metabolism (TCA cycle) |
| *SMF1* | 1,41 | 60,05 | Metal ion transporter |  | *ALR1* | 1,02 | 34,74 | Metal transporter (Mg^2+^) |
| *ULI1* | 1,38 | 2,05 | Unknown (UPR) |  | *GRE2* | 1,02 | 24,22 | Glucose metab. (methylglyoxal reductase) |
| *YLR035C-A* | 1,34 | 18,73 | Retrotransposon |  | *GCV2* | 1,02 | 2,15 | Glycine catabolism |
| *ENB1* | 1,34 | 58,71 | Metal transpoter (Fe) |  | *INO4* | 1,01 | 15,2 | Lipid metabolism (Palmitoylation) |

| Name | Log_2_ fold change | Log_10_  q value | Function |  | Name | Log_2_ fold change | Log_10_  q value | | Function |
| --- | --- | --- | --- | --- | --- | --- | --- | --- | --- |
| *TCB1* | -14,86 | 32,58 | Tricalbin |  | *YGR027W-A* | -2,67 | 2,31 | | Retrotransposon |
| *TCB2* | -13,30 | 26,52 | Tricalbin |  | *YJR128W* | -2,21 | 1,38 | | Unknown |
| *TCB3* | -12,21 | 75,48 | Tricalbin |  | *YDR261C-C* | -1,77 | 3,26 | | Retrotransposon |
| *MF(ALPHA)2* | -11,59 | 19,91 | Mating, (Pheromone) |  | *LEU1* | -1,75 | 59,96 | | Aminoacid synthesis |
| *MF(ALPHA)1* | -11,47 | 145,69 | Mating, (Pheromone) |  | *YIG1* | -1,65 | 1,39 | | Glucose metabolism |
| *IMD2* | -10,23 | 114,9 | Other (GTP synthesis) |  | *HMLALPHA2* | -1,64 | 47,94 | | Mating |
| *YHR214C-C* | -10,11 | 12,3 | Retrotransposon |  | *RPL18B* | -1,62 | 92,29 | | Other (Ribosome) |
| *HMLALPHA1* | -9,89 | 14,08 | Mating |  | *OAC1* | -1,54 | 46,37 | | Glucose metabolism (mito IM transporter) |
| *PHO12* | -9,61 | 13,12 | Other (phosphate uptake) |  | *PGU1* | -1,52 | 1,43 | | Glucose metabolism (pectolytic enzyme) |
| *YHR214C-B* | -7,84 | 3,64 | Retrotransposon |  | *YCR041W* | -1,17 | 1,35 | | Unknown |
| *STE3* | -7,14 | 281,21 | Mating |  | *COS12* | -1,14 | 7,69 | | Protein degradation (ubiquitinylation) |
| *SAG1* | -4,54 | 0 | Mating |  | *BAT1* | -1,13 | 50,78 | | Aminoacid synthesis |
| *AFB1* | -3,66 | 48,51 | Mating (cell wall) |  |  | | |  |  |

Table S2. List of differently expressed genes, WT vs. *tcb1*Δ*2*Δ*3*Δ, with Log_2_ fold change value over two. Upper Table shows upregulated genes and lower table downregulated genes. Functional characterization as in Figure 1*B* is indicated.

**Descriptions for Supporting Datasets:**

Dataset S1 (separate file). Raw RNA sequencing data, WT vs *tcb1*Δ*2*Δ*3*Δ*,* of small (RPS) and large (RPL) ribosomal subunit genes.

Dataset S2 (separate file). Raw RNA sequencing data, WT vs *tcb1*Δ*2*Δ*3*Δ*,* of 47 heat shock genes. Genes are sorted as in Quon et al., 2022 Figure 5B (24). Upregulated genes are highlighted in red and downregulated genes in blue.

Dataset S3 (separate file). Raw RNA sequencing data, WT vs *tcb1*Δ*2*Δ*3*Δ*,* of 60 HOG-regulated genes that were shown to be upregulated in *Δ-s-tether* (24). Genes are sorted as in Quon et al., 2022 - Figure 5C (24). Upregulated genes are highlighted in red and downregulated genes in blue.

Dataset S4 (separate file). Raw RNA sequencing data, WT vs *tcb1*Δ*2*Δ*3*Δ*,* of 51 UPR-regulated genes that were shown to be mostly upregulated in *Δ-s-tether* (24). Genes are sorted as in Quon et al., 2022 - Figure 5A (24). Upregulated genes are highlighted in red and downregulated genes in blue.

Dataset S5 (separate file). Complete and raw RNA sequencing dataset of WT vs *tcb1*Δ*2*Δ*3*Δ*.* Upregulated genes are highlighted in red and downregulated genes in blue.
